# Physical and psychological reconditioning in long COVID syndrome: Results of an out-of-hospital exercise and psychological - based rehabilitation program^☆^

**DOI:** 10.1016/j.ijcha.2022.101080

**Published:** 2022-07-16

**Authors:** Silvia Compagno, Stefano Palermi, Valentina Pescatore, Erica Brugin, Marzia Sarto, Ruggero Marin, Valli Calzavara, Manuele Nizzetto, Moreno Scevola, Accurso Aloi, Alessandro Biffi, Carlo Zanella, Giovanni Carretta, Silvia Gallo, Franco Giada

**Affiliations:** a Cardiovascular Rehabilitation and Sports Medicine Service, Noale - Venice, Italy; b Federico II University Hospital, Naples, Italy; c Sub-Intensive Care COVID Unit, Pneumology Service, Dolo - Venice, Italy; d Non-critical COVID Area, Internal Medicine Service, Dolo - Venice, Italy; e Med-Ex, Medicine & Exercise, Medical Partner Scuderia Ferrari, Rome, Italy; f Medical Direction, Azienda ULLS 3 Serenissima, Venice, Italy

**Keywords:** Long covid syndrome, Multidisciplinary rehabilitation, Psychological management, Physical exercise training

## Abstract

**Background:**

Long Covid Syndrome (LCS) is used to describe signs and symptoms that continue or develop after acute COVID-19 infection. Natural history and treatment of this syndrome are still poorly understood, even if evidences suggest the potential role of physical rehabilitation in improving symptoms in these patients.

**Aim of the study:**

The aim of the present study was to evaluate effectiveness, safety and feasibility of an out-of-hospital multidisciplinary rehabilitation (MDR) program, based both on physical and psychological reconditioning, in reducing symptoms and improving physical fitness and psychological parameters in patients with LCS.

**Methods:**

Thirty consecutive patients with LCS (18 males, mean age 58 years) underwent an accurate medical screening process including anthropometric and muscular strength evaluation, cardiopulmonary exercise test, quality of life (QoL) and psychological appraisal before and after a MDR program.

**Results:**

At baseline, all LCS patients were strongly symptomatic and showed severe impairments in physical performance, QoL and psychological parameters. No adverse effects and dropouts were observed during the exercise training sessions. After the MDR program, COVID-19 residual symptoms significantly decreased, and significant improvements in upper and lower limb muscular strength, cardiopulmonary parameters, perceived physical and mental health, depression and anxiety were observed.

**Conclusions:**

The present study confirms the severe physical and psychological impairment of patients with LCS and suggests that a MDR program is effective, safe and feasible in these patients and could promote their physical and psychological recovery.

## 1 Introduction

COVID-19 has resulted in a global pandemic and in a huge public health crisis [1]. Clinical characteristics of COVID-19 infection vary from a mild asymptomatic state, to a severe illness with respiratory dysfunction, thrombotic complications and multiorgan failure, especially in patients with pre-existing cardiovascular, respiratory or metabolic chronic diseases [2], [3]. Recent evidences suggest the emergence of a novel syndrome known as Long Covid Syndrome (LCS), a term used to describe signs and symptoms that continue or develop over time after acute COVID-19 infection, and that may take many months to resolve [4], [5], [6]. Researchers report that almost all hospitalized patients, 60 days after the end of acute COVID-19 infection, still present at least one symptom, and half of them experience three or more residual symptoms, such as fatigue, headache, attention disorders, and dyspnea [7], [8]. However, differently from what it could be assumed, this syndrome could also affect non-hospitalized patients.

Treatment of LCS is not well defined, given the fact that, until today, no drug therapy has been shown to improve symptomatology. Various guidelines focus on treating and managing LCS, but no agreement has been reached about the best therapeutic strategy [5]. Physical rehabilitation has been proposed as a valid approach in these patients [9], [10], [11], [12], [13], [14], and international guidelines suggest to not underestimate the weight that psychological management could have in patients with LCS [11], [15]. However, there is currently a lack of data in literature about the contemporary effectiveness of physical and psychological reconditioning in these patients. Therefore, the aim of the present paper was to evaluate effectiveness, safety and feasibility of an out-of-hospital, multidisciplinary rehabilitation (MDR) program, based both on physical and psychological reconditioning, in relieving symptoms and improving physical and psychological parameters in patients with LCS.

## 2 Methods

### 2.1 Study protocol

Thirty consecutive patients with LCS followed by Pneumology Service of Dolo – Venice, Italy were enrolled in the study. The inclusion criteria were: age ≥18 years; previous diagnosis of COVID-19 infection according to World Health Organization definition [16]; presence of symptoms continuing 4 or more weeks after the end of COVID-19 infection. Exclusion criteria were: orthopedic limitations; psychiatric or neurological disorders; any other cardiovascular contraindication to exercise testing and training. The study was carried on in accordance with the principles of the Declaration of Helsinki. Moreover, following the applicable legal regulations and the Code of Medical Ethics, subjects were duly informed on the risks, benefits and stress deriving from the physical exercise and signed an informed consent.

All the enrolled patients underwent an accurate medical screening process including rest electrocardiogram and transthoracic echocardiogram, body composition and muscular strength evaluation, cardiopulmonary exercise test (CPET), psychological and quality of life (QoL) assessment, before and after the MDR program, at the Cardiovascular Rehabilitation and Sports Medicine Service of Noale Hospital, Venice, Italy. LCS symptoms were extracted from medical records of the Pneumology Service that cared the patients. The MDR program, including both physical training and psychological treatment, was conducted by a multidisciplinary team including physical trainers, nurses, psychologists, cardiologists and sport medicine physicians. A general treatment plan was established by the multidisciplinary team and adapted for each patient, according to its clinical presentation.

The outcome of the study was to evaluate effectiveness, safety and feasibility of the MDR program. Effectiveness was measured through the improvement of the body composition, muscular strength, cardiopulmonary and psychological parameters. Safety and feasibility were measured respectively through the number of adverse cardiovascular events occurring during the training session and through the drop-out rate from the program itself

### 2.2 Body composition evaluation

All the measurements were performed in a room with a standardized temperature (21 °C), by the same trained examiner. Body weight was measured in underwear to the nearest 0.1 kg (Tanita Corporation, Japan). Height was measured with a stadiometer to the nearest 0.5 cm. Both measurements were used to calculate Body Mass Index (BMI, Kg/m^2^). Fat Mass (FM, %) and Fat Free Mass (FFM, %) were calculated through the measurement of tricep, subscapular, bicep and iliac crest skinfold thickness, using a caliper (Best K-501, Trystom, Czech Republic), and following the guidelines of the International Society for the Advancement in Kinanthropometry [17]. The equations used to estimate percentages of FM and FFM were those of Durning and of Siri, respectively [18], [19]. Muscular mass (MM, Kg) was calculated by right upper arm circumference measurement together with tricep skinfold thickness, with the formula used by Heymsfield et al. [20].

### 2.3 Muscular strength evaluation

Upper limbs muscular strength was assessed through handgrip test, while lower limbs muscular strength with leg press test. The handgrip test was performed using a digital handgrip dynamometer (Lafayette instrument company, USA), according to the standard procedures recommended by the American Society of Hand Therapists [21]. The one-repetition maximum (1-RM) for the leg press, that is the maximal weight an individual can lift for only one repetition with correct technique, was estimated using a leg press machine (Technogym, Rotterdam) following the protocol described by Kraemer et al. [22].

### 2.4 Cardiopulmonary assessment

The cardiopulmonary status was investigated through a cycle ergometer cardiopulmonary exercise test (CPET). Each patient underwent a standardized, graded exercise test using an Ebike with control terminal PC cycle ergometer (GE Medical System, Germany), with Vyntus CPX metabolic analysis system (Vyaire medical, USA), and with breath-by-breath measures of ventilation and gas exchange. Each test was performed applying a personalized ramp protocol set to achieve peak of exercise in 10 ± 2 min, while a respiratory exchange ratio (RER) >1.10 was used to consider the test as maximal [23], [24].

### 2.5 QoL assessment

QoL was assessed using the Short term Form 36 (SF-36) questionnaire, which contains nine scale for assessing physical and psychosocial domain parameters [25]. Patients must rate their current state of health on a scale of 0 (worst possible health) to 100 (best health).

### 2.6 Psychological assessment

Anxiety and depression were assessed through Zung scales. Both the Zung Self-Rating Depression Scale and Zung Self-Rating Anxiety Scale are 20-items self-report questionnaires, ranging from 20 to 80 [26], [27]. After this self-valuation process, an expert psychologist confirmed the diagnosis.

### 2.7 Physical training program

Training sessions at the hospital gym were carried out following the indication of American College of Sports Medicine [28]. Training program included 3 training sessions per week of 90 min duration. Each training session begun with 10 min of a mix of warm up, and was followed by a 45-minute endurance training: 5 min of low-intensity warm up; 35 min of continuous moderate-intensity training (corresponding to 60–80% of VO2 peak registered during CPET); 5 min of low intensity cool down. Endurance training was carried out with the use of cardio machines, such as cycle ergometer and treadmill (Technogym, Rotterdam). Aerobic exercise was followed by 20 min of resistance strength training, conducted at a variable load of 30–50% of the 1-RM, with the use of compressed-air isotonic machines (pectoral machine, lower-back, leg press, leg extension, adductor machine, deltoids press; Technogym, Rotterdam). At the end of the training session, 5 min of stretching activities were performed. Patient safety during training was ensured by strict monitoring peripheral arterial oxygen saturation, blood pressure and telemetric electrocardiogram.

### 2.8 Psychological treatment

Psychosocial treatment was performed through 4 psychological interviews. The psychological treatment was tailored to the specific symptoms of patients and was based on cognitive behavioral therapy and eye movement desensitization and reprocessing therapy. Individual relaxation techniques, such as muscular relaxation, body-scan, breath control, and imaginative relaxation were also performed [29]. Moreover, multidisciplinary counselling was performed through grouped and individualized health education meetings, during which patients were educated about their condition, and to adopt active lifestyle modification, such as improving physical activity levels, eating healthier and stop smoking.

### 2.9 Statistical analysis

By means of Shapiro-Wilk test we were able to verify that all outcome parameters were normal distributed. Quantitative variables were summarized as mean (m) and standard deviation (SD), while categorical variables as absolute values (n) and percentages (%). The null hypothesis of the study was that there were no differences between outcome parameters before and after the MDR program. For continuous variables, changes from pre- to post MDR program was tested with paired t tests, while Fisher’s exact test was used to compare categorical variables. A p-value < 0.05 was considered statistically significant. The analysis has been carries out with STATA software (STATA, v.8, Italy).

## 3 Results

Clinical characteristics of study population are reported in Table 1. We analyzed 30 consecutive patients with LCS, between April 2021 and November 2021, after a mean of 3 months (range 1–6 months) from the resolution of acute COVID-19 infection. During acute COVID-19 infection, about 16% of patients needed mechanical ventilation and was treated in Intensive Care Unit, while 53.3% needed only non-invasive oxygen supply, and 30% didn’t need hospital admission. The mean age was 58.37 years, and 60% were males. Most of them were overweight, since mean BMI was 29.2 kg/m^2^. Thirty-three percent had hypertension and about half was actual smoker. Resting ECG and echocardiogram excluded any Covid-19 related cardiac involvement. During the MDR program, each LCS patients underwent 4 psychological interviews, and 13 physical training sessions on average (range 8–20).

**Table 1:** Baseline clinical characteristics of study population (n = 30).

Age – years (mean ± SD)	58.37 ± 11.6
Male, n (%)	18 (60%)
Comorbidities, n (%)	
Hypertension	10 (33.3%)
Diabetes	3 (10%)
Hypercholesterolemia	9 (30%)
None	8 (26.6%)
Smoking habit, n (%)	
Actual	16 (53.3%)
Past	7 (23.3%)
Never	7 (23.3%)
Time between recovery from acute COVID-19 infection to MDR – months mean (range)	3 (1–6)

*Site of treatment for acute COVID-19 infection*	
Hospital departments, n (%)	16 (53.3%)
Intensive Care Unit, n (%)	5 (16.6%)
Home, n (%)	9 (30%)

*Respiratory support for acute COVID-19 infection*	
Oxygen supply, n (%)	16 (53.3%)
Mechanical ventilation, n (%)	5 (16.6%)
None, n (%)	9 (30%)

MDR = multidisciplinary rehabilitation.

### 3.1 Efficacy of the MDR program

**Fig. 1:**
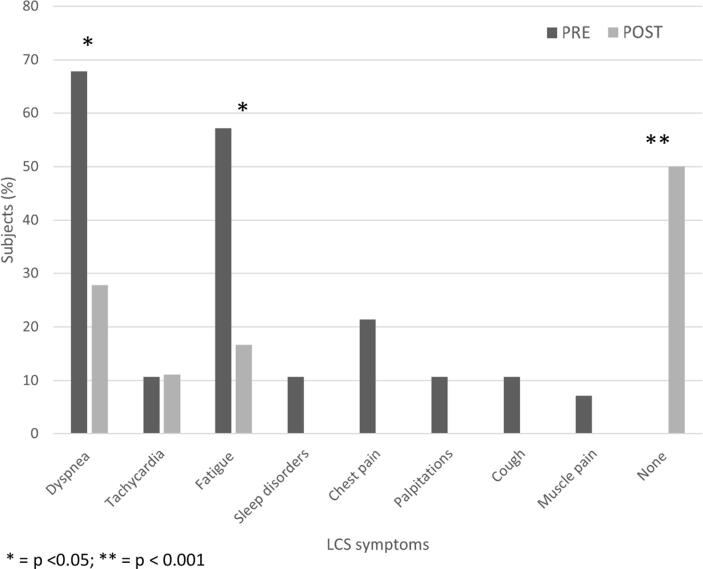
Symptoms PRE and POST the rehabilitation program in patients with Long Covid Syndrome *p < 0.05; ^**^p < 0.001.

At baseline, all patients suffered from at least one COVID-19 residual symptom, with dyspnea and fatigue as the most represented; after the MDR program, all symptoms decreased significantly, with 50% of patients feels no symptoms at all (Fig. 1). Before the MDR program, patients had an unfavorable body composition with high FM percentage and reduced muscular strength values; at the end of the MDR program, the mild improvements in body composition parameters did not reach the statistically significance, while upper and lower limb muscular strength values improved significantly (Table 2). All CPET resulted negative from ischemic point of view, and no variations in arterial SpO_2_ between resting and peak values were observed. Cardiopulmonary fitness was reduced at baseline, with the mean VO2 peak corresponding only to 73% of predicted value. After the MDR program, significant improvements in VO2 peak and peak work rate were observed (Table 3). At enrollment patients showed a depressed QoL, with low physical and mental domain values, and high levels of depression and anxiety; however, at the end of the study period, almost all physical and mental domain values improved significantly, with a contemporary reduction of anxiety and depression scores (Table 4).

**Table 2:** Body composition and muscular strength PRE and POST the rehabilitation program.

	PRE	POST
BMI - Kg/m^2^ (mean ± SD)	29.20 ± 4.3	29.33 ± 4
Muscle mass – Kg (mean ± SD)	32.29 ± 8.4	33.19 ± 9.9
Fat mass (%)	28.14	27.75
Free fat mass (%)	71.86	72.25
Handgrip strength – Kg (mean ± SD)	39.04 ± 11.1	45.12 ± 11.3*
Leg Press strength – Kg (mean ± SD)	198.38 ± 50.4	225.81 ± 51.9^**^

BMI = body mass index *p < 0.05; ^**^p < 0.001.

**Table 3:** Cardiopulmonary values PRE and POST the rehabilitation program.

	PRE	POST
Peak work rate – Watt (mean ± SD)	113.17 ± 41	133.91 ± 43.3^**^
satO2 baseline (%)	96.82	97.17
satO2 peak (%)	95.60	96.52
VO2 peak – mL/Kg/min (mean ± SD)	17.53 ± 5.2	20 ± 4.4*
VO2 peak (% predicted)	73.43	82.54*
Anaerobic threshold – mL/Kg/min (mean ± SD)	13.38 ± 4.5	14.74 ± 3.4
VE/VCO2 slope (mean ± SD)	30.84 ± 4.7	29.48 ± 4.2

SatO2 baseline = baseline arterial O2 saturation; satO2 peak = peak arterial O2 saturation; VO2 peak = absolute peak VO2; VO2 peak (% predicted) = percentage of VO2 peak predicted value.

*p < 0.05; ^**^p < 0.001.

**Table 4:** Quality of life and psychological evaluation PRE and POST the rehabilitation program.

	PRE	POST
*Physical domain (SF-36) (mean ± SD)*		
Physical functioning	57.95 (±22.4)	82.73 (±18.3)*
Role Limitation due to Physical Health	17.95 (±32.8)	54.54 (±45.4)^**^
Energy/Fatigue	43.64 (±15.7)	60.55 (±20)*
Bodily Pain	53.16 (±26.5)	67.18 (±29.2)*
General Health	53.63 (±17.1)	63.40 (±23.4)*

*Mental domain (SF-36) (mean ± SD)*		
Role Limitation due to Emotional Health	25.72 (±37)	66.63 (±46)^**^
Mental Health	60.86 (±18.7)	72.40 (±17.4)
Social Functioning	53.22 (±24.2)	73.77 (±27.9)*
Health Changes	13.63 (±14.9)	46.59 (±33)^**^
Self-rating depression scale (Zung) (mean ± SD)	40.45 (±8.6)	36.27 (±8.5)*
Self-rating anxiety scale (Zung) (mean ± SD)	39.59 (±8.9)	34.22 (±8.5)*

*p < 0.05; ^**^p < 0.001.

### 3.2 Safety and feasibility of the MDR program

No adverse events nor dropouts were observed during the training period.

## 4 Discussion

LCS is an increasingly recognized problem facing the globally Covid 19 infected population [30]. Indeed, it has a major role in reducing QoL of affected patients, delaying the recovery of the normal activity of daily living [31]. Natural history of LCS is still poorly understood since pathophysiological mechanisms are not yet completely clear [9]. Its pathogenesis is probably multi-factorial and more than one mechanism may be implicated, even if prolonged inflammation seems to have a key role [32]. Physical rehabilitation has been theorized and proposed as a valid therapeutic strategy for this syndrome [11]. To the authors’ best knowledge, this is the first study evaluating the contemporary effectiveness of a MDR program, based at the same time both on physical and psychological reconditioning, in reducing symptoms and improving physical and psychological parameters in patients with LCS.

### 4.1 Efficacy of the MDR program

In line with previous studies, all our patients suffered from at least one COVID-19 residual symptoms [7], [8], [14]: dyspnea and fatigue were the two most represented, but also tachycardia, sleep disorders, chest pain and palpitations were quite frequent. As a main result, we observed a significant reduction of COVID-19 residual symptoms: 50% of subjects were free-of-symptoms at the end of the study period, and rate of dyspnea and fatigue significantly crashed. This is a big result, since persisting symptoms in LCS are the leading cause of disability and reduced QoL, and thus their reduction should be a main target of every proposed therapy [4], [11].

#### 4.1.1 Body composition and muscular strength

Almost all LCS patients were overweight and showed an high percentage of body fat, with a reduced muscular mass and strength. Indeed, an independent deleterious impact of COVID-19 infection on muscular strength has been already described [33], [34]. In the present study, values of upper and lower limb muscular strength improve significantly after the MDR program: probably the synergy between endurance and resistance training can explain these results. This relevant muscular fitness improvement can have big public health consequences [35], [36].

#### 4.1.2 Cardiopulmonary fitness

All our patients with LCS showed at baseline low cardiopulmonary fitness at CPET evaluation, probably due to a peripheral deficit, as already shown by Clavario et al. [33]. Most of CPET parameters, however, significantly improved after the MDR program, with a greater improvement of VO2 peak, likely in relation to the above increase in muscular strength. This improvement is very significant because it has been demonstrated that a low VO2 peak is related with some LCS symptoms, such as dyspnea and fatigue [37]; moreover, data from literature show that increase of VO2 peak are associated with improvement of several clinical outcomes [38]. Indeed, CPET is stated in the literature as one of the crucial tests in LCS patients evaluation and exercise prescription [39], [40].

#### 4.1.3 QoL status

Patients with LCS showed a poor QoL at baseline; after the MDR, we highlighted high improvement in almost all physical and mental related domains of QoL.

#### 4.1.4 Psychological status

In the present study patients with LCS showed high values of anxiety, depression and sleep disturbances, in line with recent reports [41], [42], [43], [44], [45]. After the MDR program we highlighted high improvements in self-related anxiety and depression levels. Thus, cognitive behavioral therapy was effective in treating patients with LCS, as already shown by Ferrario et al. [29]. Probably the synergy between psychological management, multidisciplinary counseling and physical exercise acted a major role in these results [46].

### 4.2 Safety and feasibility of the MDR program

We didn’t observe side effects, nor clinical complications during the MDR program, as already shown in previous report [47], [48], [49]. Patients showed good compliance to training sessions and psychological management, and no drop-outs were reported during the study period.

### 4.3 Limits of the study

Our study suffers from some limitations. First, our sample size was composed only by 30 patients, even if other studies on the same topic have a similar sample size [12], [14], [50]. Secondly, we had not a control group, and we did not differentiate the impact of specific treatment measures, such as physical training and psychological interventions, but rather focused on the overall effects of the multidisciplinary rehabilitation strategy. LCS symptoms were not measured with validated questionnaire, but rather were derived from medical records of patients. We decided to exclude to MDR program subjects who could have contraindication to exercise training, and this exclusion may result into a less representative sample of LCS patients. Moreover, we did not perform a real nutritional management of these patients, and this is probably the reason, along with the short study period, of the lack of significant improvements in body composition after the MDR program. However, this could be a target for future studies, since malnutrition, other than a key cardiovascular risk factor, can affect duration of hospitalization, impair the immune system, and contribute to muscle wasting in patients with LCS. Lastly, we have no data about the long-term follow up of these patients, so that we are not sure if our promising results can be maintained over time.

### 4.4 Conclusions

The present study confirms the severe global impairment of patients with LCS, and suggest that an out-of-hospital, exercise and psychological-based MDR program is safe and feasible in these patients,and could reduce residual symptoms and promote physical and psychological recovery. Further studies should confirm these results in larger cohort of patients, and with a long-term follow-up, with a possible focus on the cognitive problems that seem to be highly prevalent and long-lasting in these patients.

## Declaration of Competing Interest

The authors declare that they have no known competing financial interests or personal relationships that could have appeared to influence the work reported in this paper.

## References

[1] WHO Coronavirus (COVID-19) | WHO Coronavirus (COVID-19) Dashboard With Vaccination Data.

[2] Singhal T. (2020). A review of coronavirus disease-2019 (COVID-19). Indian J. Pediatr..

[3] Flaherty G.T., Hession P., Liew C.H., Lim B.C.W., Leong T.K., Lim V., Sulaiman L.H. (2020). COVID-19 in adult patients with pre-existing chronic cardiac, respiratory and metabolic disease: a critical literature review with clinical recommendations. Trop. Dis. Travel Med. Vacc..

[4] Taribagil P., Creer D., Tahir H. (2021). “Long COVID” syndrome. BMJ Case Rep..

[5] Overview | COVID-19 Rapid Guideline: Managing COVID-19 | Guidance | NICE.

[6] Petersen M.S., Kristiansen M.F., Hanusson K.D. (2021). Long COVID in the faroe islands: a longitudinal study among nonhospitalized patients. Clin. Infect. Dis. Off. Publ. Infect. Dis. Soc. Am..

[7] Carfì A., Bernabei R., Landi F. (2020). Persistent symptoms in patients after acute COVID-19. JAMA.

[8] Lopez-Leon S., Wegman-Ostrosky T., Perelman C., Sepulveda R., Rebolledo P.A., Cuapio A., Villapol S. (2021). More than 50 long-term effects of COVID-19: a systematic review and meta-analysis. Sci. Rep..

[9] Yong S.J. (2021). Long COVID or post-COVID-19 syndrome: putative pathophysiology, risk factors, and treatments. Infect Dis (Auckl).

[10] Sisó-Almirall A., Brito-Zerón P., Conangla Ferrín L., Kostov B., Moragas Moreno A., Mestres J., Sellarès J., Galindo G., Morera R., Basora J., Trilla A., Ramos-Casals M. (2021;18,). Long Covid-19: proposed primary care clinical guidelines for diagnosis and disease management. Int. J. Environ. Res. Public Heal.

[11] Barker-Davies R.M., O'Sullivan O., Senaratne K.P.P., Baker P., Cranley M., Dharm-Datta S., Ellis H., Goodall D., Gough M., Lewis S., Norman J., Papadopoulou T., Roscoe D., Sherwood D., Turner P., Walker T., Mistlin A., Phillip R., Nicol A.M., Bennett A.N., Bahadur S. (2020). The Stanford Hall consensus statement for post-COVID-19 rehabilitation. Br. J. Sports Med..

[12] Puchner B., Sahanic S., Kirchmair R., Pizzini A., Sonnweber B., Wöll E., Mühlbacher A., Garimorth K., Dareb B., Ehling R., Wenter J., Schneider S., Brenneis C., Weiss G., Tancevski I., Sonnweber T., Löffler-ragg J. (2021). Beneficial effects of multi-disciplinary rehabilitation in postacute COVID-19: an observational cohort study. Eur. J. Phys. Rehabil. Med..

[13] Tozato C., Ferreira B.F.C., Dalavina J.P. (2021). Cardiopulmonary rehabilitation in post-COVID-19 patients: case series. Rev. Bras. Ter. Intensiva.

[14] Barbara C., Clavario P., De Marzo V. (2022). Effects of exercise rehabilitation in patients with long COVID-19. Euro. J. Prevent. Cardiol..

[15] Ambrosetti M., Abreu A., Cornelissen V. (2021). Delphi consensus recommendations on how to provide cardiovascular rehabilitation in the COVID-19 era. Eur J Prev Cardiol..

[16] WHO Coronavirus (COVID-19) | WHO Coronavirus (COVID-19) Case Definitions.

[17] Silva V.S., Vieira F. (2020). International Society for the Advancement of Kinanthropometry (ISAK) Global: international accreditation scheme of the competent anthropometrist. Rev. Bras. Cineantropom. Desempenho. Hum..

[18] Durnin J.V.G.A., Womersley J. (1974). Body fat assessed from total body density and its estimation from skinfold thickness: measurements on 481 men and women aged from 16 to 72 years. Br. J. Nutr..

[19] Guerra R.S., Amaral T.F., Marques E., Mota J., Restivo M.T. (2010). Accuracy of Siri and Brozek equations in the percent body fat estimation in older adults. J. Nutr. Health Aging..

[20] Heymsfield S.B., McManus C., Smith J. (1982). Anthropometric measurement of muscle mass: revised equations for calculating bone-free arm muscle area. Am. J. Clin. Nutr..

[21] Fess E. (1981). Clinical assessment recommendations. Am. Soc. Hand Ther..

[22] Kraemer W.J., Adams K., Cafarelli E. (2002). American College of Sports Medicine position stand. Progression models in resistance training for healthy adults. Med. Sci. Sports Exerc..

[23] Herdy A.H., Ritt L.E.F., Stein R. (2016). Cardiopulmonary exercise test: background, applicability and interpretation. Arq. Bras. Cardiol..

[24] Weisman I.M., Zeballos R.J. (2002).

[25] Ware J.E.J., Sherbourne C.D. (1992). The MOS 36-item short-form health survey (SF-36). I. Conceptual framework and item selection. Med. Care.

[26] Zung W.W.K. (1971). A rating instrument for anxiety disorders. Psychosomatics.

[27] Zung W.W.K. (1965). A self-rating depression scale. Arch. Gen. Psychiatry.

[28] Wolters Kluwer ed., ACSMs Guidelines for Exercise Testing and Prescription. Eleventh.

[29] Rossi Ferrario S., Panzeri A., Cerutti P., Sacco D. (2021). The psychological experience and intervention in post-acute COVID-19 inpatients. Neuropsychiatr. Dis. Treat..

[30] Hoffer E.P. (2021). Long COVID: does it exist? what is it? we can we do for sufferers?. Am. J. Med..

[31] Poudel A.N., Zhu S., Cooper N., Roderick P., Alwan N., Tarrant C., Ziauddeen N., Yao G.L., Mitra P. (2021). Impact of Covid-19 on health-related quality of life of patients: a structured review. PLoS ONE.

[32] Maltezou H.C., Pavli A., Tsakris A. (2021). Post-COVID syndrome: an insight on its pathogenesis. Vaccines.

[33] Del Brutto O.H., Mera R.M., Pérez P. (2021). Hand grip strength before and after SARS-CoV-2 infection in community-dwelling older adults. J. Am. Geriatr. Soc..

[34] Tuzun S., Keles A., Okutan D., Yildiran T., Palamar D. (2021). Assessment of musculoskeletal pain, fatigue and grip strength in hospitalized patients with COVID-19. Eur. J. Phys. Rehabil. Med..

[35] McGrath R.P., Kraemer W.J., Snih S.A., Peterson M.D. (2018). Handgrip strength and health in aging adults. Sports Med..

[36] Buckner S.L., Dankel S.J., Bell Z.W., Abe T., Loenneke J.P. (2019). The association of handgrip strength and mortality: what does it tell us and what can we do with it?. Rejuven. Res..

[37] Aparisi Á., Ybarra-Falcón C., García-Gómez M., Tobar J., Iglesias-Echeverría C., Jaurrieta-Largo S., Ladrón R., Uribarri A., Catalá P., Hinojosa W., Marcos-Mangas M., Fernández-Prieto L., Sedano-Gutiérrez R., Cusacovich I., Andaluz-Ojeda D., de Vega-Sánchez B., Recio-Platero A., Sanz-Patiño E., Calvo D., Baladrón C., Carrasco-Moraleja M., Disdier-Vicente C., Amat-Santos I.J., San Román J.A. (2021). Exercise ventilatory inefficiency in post-COVID-19 syndrome: insights from a prospective evaluation. J. Clin. Med..

[38] Mohamed A.A., Alawna M. (2020). Role of increasing the aerobic capacity on improving the function of immune and respiratory systems in patients with coronavirus (COVID-19): a review. Diabetes Metab. Syndr..

[39] Ahmed I. (2020). COVID-19 - does exercise prescription and maximal oxygen uptake (VO(2) max) have a role in risk-stratifying patients?. Clin. Med..

[40] Dorelli G., Braggio M., Gabbiani D. (2021). Importance of cardiopulmonary exercise testing among subjects recovering from covid-19. Diagnostics.

[41] Rajkumar R.P. (2020). COVID-19 and mental health: a review of the existing literature. Asian J. Psychiatr..

[42] G. Orrù, D. Bertelloni, F. Diolaiuti et al., Long-COVID Syndrome? A Study on the Persistence of Neurological, Psychological and Physiological Symptoms. Healthc (Basel, Switzerland) 9 (2021). doi: 10.3390/healthcare9050575. 10.3390/healthcare9050575 PMC8152255 34068009

[43] Naidu S.B., Shah A.J., Saigal A., Smith C., Brill S.E., Goldring J., Hurst J.R., Jarvis H., Lipman M., Mandal S. (2021). The high mental health burden of “Long COVID” and its association with on-going physical and respiratory symptoms in all adults discharged from hospital. Eur. Respir. J..

[44] Shanbehzadeh S., Tavahomi M., Zanjari N., Ebrahimi-Takamjani I., Amiri-arimi S. (2021). Physical and mental health complications post-COVID-19: Scoping review. J. Psychosom. Res..

[45] Houben-Wilke S., Goërtz Y.MJ., Delbressine J.M., Vaes A.W., Meys R., Machado F.VC., van Herck M., Burtin C., Posthuma R., Franssen F.ME., Vijlbrief H., Spies Y., van 't Hul A.J., Spruit M.A., Janssen D.JA. (2022). The impact of long COVID-19 on mental health: observational 6-month follow-up study. JMIR Ment Health..

[46] Chekroud S.R., Gueorguieva R., Zheutlin A.B., Paulus M., Krumholz H.M., Krystal J.H., Chekroud A.M. (2018). Association between physical exercise and mental health in 1·2 million individuals in the USA between 2011 and 2015: a cross-sectional study. lancet Psychiatry.

[47] Clavario P., De Marzo V., Lotti R. (2021). Cardiopulmonary exercise testing in COVID-19 patients at 3 months follow-up. Int. J. Cardiol..

[48] Martin I., Braem F., Baudet L., Poncin W., Fizaine S., Aboubakar F., Froidure A., Pilette C., Liistro G., De Greef J., Yildiz H., Pothen L., Yombi J.-C., Belkhir L., Reychler G. (2021). Follow-up of functional exercise capacity in patients with COVID-19: It is improved by telerehabilitation. Respir. Med..

[49] Demeco A., Marotta N., Barletta M. (2020). Rehabilitation of patients post-COVID-19 infection: a literature review. J. Int. Med. Res..

[50] Curci C., Negrini F., Ferrillo M., Bergonzi R., Bonacci E., Camozzi D.M., Ceravolo C., De franceschi S., Guarnieri R., Moro P., Pisano F., Sire A.D. (2021). Functional outcome after inpatient rehabilitation in postintensive care unit COVID-19 patients: findings and clinical implications from a real-practice retrospective study. Eur. J. Phys. Rehabil. Med..

